# Forest Cover Classification by Optimal Segmentation of High Resolution Satellite Imagery

**DOI:** 10.3390/s110201943

**Published:** 2011-02-01

**Authors:** So-Ra Kim, Woo-Kyun Lee, Doo-Ahn Kwak, Greg S. Biging, Peng Gong, Jun-Hak Lee, Hyun-Kook Cho

**Affiliations:** 1 Division of Environmental Science and Ecological Engineering, Korea University, Seoul 136-701, Korea; E-Mails: allwhile@korea.ac.kr (S.-R.K.); tulip96@korea.ac.kr (D.-A.K.); 2 Department of Environmental Science, Policy and Management, University of California at Berkeley, Mulford Hall, Berkeley, CA 94720, USA; E-Mails: biging@berkeley.edu (G.S.B.); penggong@berkeley.edu (P.G.); jhlee@berkeley.edu (J.-H.L.); 3 Division of Forest Resources Information, Korean Forest Research Institute, Seoul 136-012, Korea; E-Mail: hcho@forest.go.kr (H.-K.C.)

**Keywords:** digital forest cover map, high resolution, satellite image, pixel-based classification, segment-based classification

## Abstract

This study investigated whether high-resolution satellite imagery is suitable for preparing a detailed digital forest cover map that discriminates forest cover at the tree species level. First, we tried to find an optimal process for segmenting the high-resolution images using a region-growing method with the scale, color and shape factors in Definiens^®^ Professional 5.0. The image was classified by a traditional, pixel-based, maximum likelihood classification approach using the spectral information of the pixels. The pixels in each segment were reclassified using a segment-based classification (SBC) with a majority rule. Segmentation with strongly weighted color was less sensitive to the scale parameter and led to optimal forest cover segmentation and classification. The pixel-based classification (PBC) suffered from the “salt-and-pepper effect” and performed poorly in the classification of forest cover types, whereas the SBC helped to attenuate the effect and notably improved the classification accuracy. As a whole, SBC proved to be more suitable for classifying and delineating forest cover using high-resolution satellite images.

## 1. Introduction

Forest cover maps containing spatial information about tree species, age and diameter class, and density of each forest type are widely used for forest resource management. In Korea, analog forest cover maps were conventionally produced on topographic maps at a scale of 1:25,000, through visual interpretation of 1:15,000 scale aerial photographs [1]. Until the middle of the 1990s, the forest cover maps were prepared with a series of complex processes, namely digitization by visual interpretation using a mechanical plotting instrument. This approach is, however, time-consuming and labor-intensive for making forest cover maps of the entire forested area in South Korea.

A promising alternative is the direct mapping of forest cover types from high-resolution satellite images, which has the advantage of covering relatively large land areas on potentially regular repeating cycles. A number of earlier studies have used coarse spatial resolution satellite imagery for forest cover mapping [2], which allowed the classification of forest cover into broad categories, such as coniferous, deciduous, and mixed forests. While broad category forest cover classifications are useful in some instances, they are not suitable for providing detailed forest cover information according to tree species. Tree species can be identified or classified using high-resolution aerial images [3–7] and high-resolution multispectral satellite images [8–10]. Since high-resolution multispectral satellite image such as IKONOS and Quickbird image has been commercialized, researchers have classified land cover or land use patterns at a detailed level [11–14].

In Korea, researchers have also utilized high-resolution multispectral satellite image to identify several tree species and classify forest stands [15–18]. Higher spatial resolution images have been shown to reduce the proportion of mixed pixels and provide the possibility for better interpretation [11,12]. Pixel-based classification (PBC) approaches are traditional methods for image classification but suffer from several problems when applied to high spatial resolution images. Due to the shade and gaps, high spatial resolution data can also increase the spectral variation, structural composition and heterogeneity within a class, which might cause confusion among distinct land use categories [12], or may also lead to unwanted details [12]. Consequently, the accuracy assessment shows low performance, accompanied by the salt-and-pepper effect which is not suitable for the stand level forest management. To overcome such problems, some pixel-based methods have already been implemented, mainly consisting of three categories: (a) image pre-processing, such as low-pass filter and texture analysis [19,20], (b) contextual classification, such as Markov random field [21,22], and (c) post-classification processing, such as mode filtering, morphological filtering and rule-based processing [23,24]. These techniques incorporate spatial information to characterize each class using neighborhood relationships. However, despite their considerable improvement in classification, these techniques suffer significant disadvantages. First, the pre-defined neighborhood window size may favor all the land-cover types evenly since different classes reach their maximum accuracies at different pixel window sizes [25]. These processes have blur effects and cannot produce accurate results at the boundaries of different land-cover units [25].

Object-based classification (OBC) may be a good alternative to the pixel-based methods. It is useful for analyzing groups of contiguous pixels as objects instead of using the conventional PBC unit [25]. The OBC approach uses contextual relations between neighboring objects [26], shape features, such as surface area, length and width of the objects [13], form and size [27], structural composition of land use [12], and a combination of spectral and spatial information [7,14], in addition to spectral information.

OBC has been found to be suitable for segmenting and classifying urban or agricultural areas whose objects usually have regular shapes [7,12,24,28]. The assumption underlying this approach is that the land use or cover can be distinguished on the basis of the differences in the shapes and sizes [12]. Accordingly, assuming the image needs to be divided into meaningful homogeneous forest types, as defined by a forest classification scheme, the object-based image classification approach was used in this study. However, difficulties might be encountered when trying to segment complex forest stand patterns using an object-based approach because the objects (forest stands) may be quite dissimilar in size and shape, and their spatial location on the landscape given can vary from random to clustered. Therefore, a new approach needs to be applied for segmenting forest cover.

This study proposes suitable criteria for classifying Korea’s forest cover and uses objects as minimum and maximum classification units to overcome the problem of the salt-and-pepper effect resulting from pixel-based-classification methods. Segment-based classification (SBC) is applied using objects (segments) with pixel-based classification result. SBC has been considered as an alternative to classifying high-resolution satellite image [13,29,30] and has been evaluated as being suitable for preparing a polygon-based forest cover map.

This study explored the potential of high-resolution IKONOS images, in an SBC, for mapping detailed forest cover types. The IKONOS images were evaluated for identifying tree species, with the ultimate aim of providing a suitable method for the direct preparation of polygon-based forest cover maps.

## 2. Materials and Methods

### 2.1. Study Area and Materials

**Figure 1.**
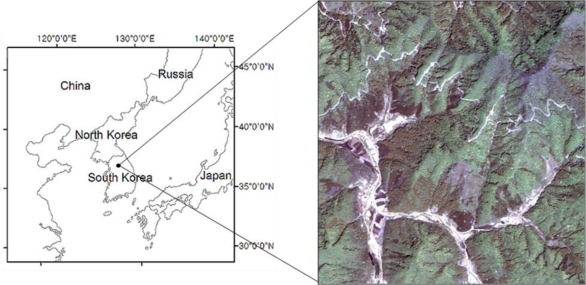
The study area with the pan-sharpened IKONOS image.

The study area was a representative rural landscape, comprised mostly of forest land, with a few agricultural and residential areas. This area represents well the typical forest cover types and topography of central Korea. *Quercus* spp. (Oak) and other deciduous tree species evenly dominated the entire area, as typically found in central Korea, with some planted coniferous stands of *Pinus koraiensis* (Korean pine), *Larix leptolepis* (Japanese larch) and *Pinus rigida* (Pitch pine) distributed at relatively lower elevations. Elevation in the study area ranged from 74 to 560 m, while about 60% of the slopes were over 20 degrees, with aspects evenly distributed in all directions. The study area was selected between 127°39′54″E, 37°29′47″N and 127°41′26″E, 37°28′26″N, covering an area of 592 ha. The corresponding portion of the IKONOS image is shown in Figure 1.

The image was acquired on 8th May, 2000, of an 11 km × 11 km area in central Korea, and used to test various classification algorithms for mapping forest types. In general, the IKONOS image was composed of four spectral bands with 4 m spatial resolution and one panchromatic band with 1 m spatial resolution. A panchromatic band with 1m spatial resolution and four multispectral bands were fused through Intensity, Hue, and Saturation (IHS) transformation with a 4-3-2 band combination for RGB color. A fused pan-sharpened image was then used for classification.

Segments, which are partial areas characterized into the same tree species, were used as the unit of the training dataset. Based on field visits and visual observations, 240 segments were selected as training areas. The segment size ranged from 25 to 7,239 m^2^ according to the tree species and stand condition, with a mean of 702 m^2^. This large range of segment sizes was attributed to the fact that the stands were not spectrally homogeneous in the high spatial resolution image, even though they can be considered naturally homogeneous. In each classification class 409 test areas were randomly and independent selected. We performed visual interpretation of the image to identify potential accuracy assessment areas, and investigated each of these areas in the field.

### 2.2. Methods

#### 2.2.1. Classification Scheme

The number and type of classes in a map are generally dependent on the information requirements and availability of certain types of remotely sensed data used for classification. To create a forest cover map, a suitable classification scheme, which has a sufficient level of detail for the intended use, is required. The goal here was to classify the forest into these individual tree species, and to prepare a detailed forest cover map. During the early growing season in the study area, tree species, especially deciduous trees, begin to grow and display different leaf and crown colors. The IKONOS image also enabled us to optically classify the forest cover for some deciduous as well as coniferous tree species. Eight classes of forest area and one class of non-forest area were used for legends in this study (Table 1).

#### 2.2.2. Classification Methods

Firstly, the IKONOS image was classified using a conventional PBC with a maximum likelihood classifier [31], using ERDAS Imagine 9.2 program. For the PBC, spectral values of red (R), green (G), blue (B) and near infrared (NIR) bands were used. Results of the PBC were used for SBC using a majority rule under different segmentation options.

##### Segmentation

For the SBC [12,13], the IKONOS image was firstly segmented using Definiens^®^ Professional 5 Program [32], which uses a region-growing segmentation approach, where the segment size is determined firstly with a scale parameter measuring the maximum possible homogeneity. The higher the value, the larger the resulting image objects [3]. Homogeneity criteria were set up using color and shape parameters, which define the total relative homogeneity of the resulting image objects. Color refers to the digital value of the resulting image objects, and shape to the textural homogeneity of the resulting image objects. The shape criterion was composed of two parameters: smoothness was used to optimize objects with regard to the smoothness of the borders, and compactness to optimize image objects with regard to their compactness [33].

The selection of the optimal combination of parameters for the segmentation is dependent on the segmentation goal and data type. In the absence of any generally accepted criteria for segmenting forest area [8], tests must be run until the appropriate segmentation parameters have been found [34].

Different pairs of weights were employed to determine the optimal criteria for segmenting forest cover (Table 2). In step I, extreme weights (0.1 or 0.9) were assigned to color and shape. In step II, the average weights (0.5) were assigned to color and shape, after which the average and extreme weights (0.1, 0.9) were assigned to smoothness. In step III, weights of 0.75 and 0.25 were given to color, with average weights given to smoothness and compactness. Different scales, from 30 to 280, were applied to each pair of weights for segmenting the study area.

##### Segment-Based Classification (SBC) Using a Majority Rule

An SBC method, utilizing the majority rule, was employed for reclassifying the segments using the results of the PBC. In this approach, the pixels of each class classified by the pixel-based method were counted using the GRID module of ArcInfo, with a particular class occupying the majority of a segment assigned to the entire segment.

#### 2.2.3. Verification of Segmentation and Classification

The classification result was verified using the independent reference unit or test area. As reference units, we employed a cluster of pixels [35]. Regarding the cluster size, the area for accuracy assessment must be larger than the spatial resolution of image [36], and that of the error of the GPS receiver, Trimble Pathfinder XR, should be within approximately ±3 m in a forested area. Therefore, we determined that an appropriate size of a cluster would be 9 × 9 pixels, which is large enough to compensate for such errors.

We used both homogeneous and majority rule for assigning tree species to the cluster and distributing the test area [37]. Based on the points randomly distributed in the study area, clusters with 9 × 9 pixels were placed such that they completely belong to a homogeneous segment and a tree species, which occupied a majority in the cluster, was assigned to the cluster.

The number of test areas in each class was established as 50 according to the rule of thumb [35] and adjusted by considering the occupation area of each class. A total of 409 test areas were established for accuracy assessment of nine classification classes. Kappa values or K-HAT statistics [27,31] were used for comparing segmentation alternatives and deciding optimal segment size. The results of PBC and SBC with the optimal segment size found in this study were compared with the error matrix and map.

## 3. Results and Discussions

### 3.1. Signature Analysis

**Figure 2.**
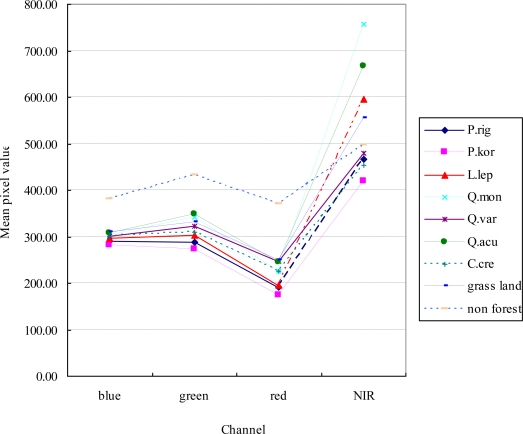
Average spectral signature of the IKONOS image for a selected training area of each class (P.rig: *Pinus rigida*, P.kor: *Pinus koraiensis*, L.lep: *Larix leptolepis*, Q.mon: *Quercus mongolica*, Q.var: *Quercus variabilis*, Q.acu: *Quercus acutissima*, C.cre: *Castanea crenata*, grass land, non forest).

Figure 2 shows the average signature diagram for the various forest type classes of training areas obtained from the IKONOS image. While the first three channels show only modest spectral differences between the classes, the fourth near the infrared channel shows relatively good spectral separation between classes [10]. Infrared has been considered a useful band for separating coniferous and deciduous trees [31,38]. As in previous works [31,38], the coniferous and deciduous trees of our study also showed distinctive differences in the infrared band. Deciduous trees had relatively higher infrared spectral values than coniferous trees. Japanese Chestnut (*Castanea crenata*) had a lower spectral value than other deciduous trees because its leaves are not fully developed by early May when the scene was acquired. Pitch pine (*Pinus rigida*) and Korean pine (*Pinus koraiensis*) had similar mean spectral characteristics.

### 3.2. Optimal Segment size

**Figure 3.**
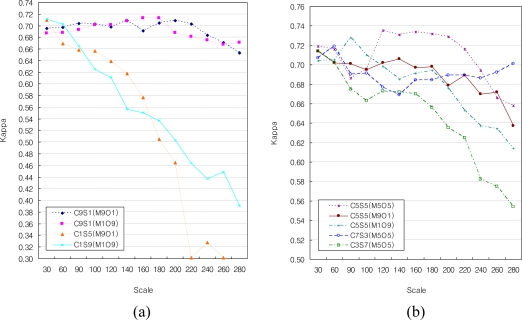
The kappa values of SBCs: **(a)** strong weights for color or shape, and **(b)** medium weights for color and shape.

Figure 3(a) shows the kappa values of the classification, with strong weights assigned to color or shape (Step I). C_9_S_1_(M_9_O_1_) and C_9_S_1_(M_1_O_9_) showed a similar accuracy when strong weights were applied for color and compactness. The accuracies of the SBCs increased as the scale parameter increased up to around 140, but then slightly decreased. The accuracy of the classifications using strongly weighted colors was relatively high with large scale parameters, as they segmented objects largely according to the spectral value, despite the large scale parameter.

C_1_S_9_(M_9_O_1_) and C_1_S_9_(M_1_O_9_), with strong weights for shape, had similar accuracies to the C_9_S_1_(M_9_O_1_) and C_9_S_1_(M_1_O_9_) with low scale parameters up to 100. However, the accuracy of C_1_S_9_(M_9_O_1_) and C_1_S_9_(M_1_O_9_) abruptly decreased as the scale parameter increased over 100. Therefore, the accuracy of C_1_S_9_(M_9_O_1_) and C_1_S_9_(M_1_O_9_), which are strongly weighted for shape, was low with large scale parameters. In C_1_S_9_(M_9_O_1_) and C_1_S_9_(M_1_O_9_), the shape strongly influenced the segmentation, and segments having similar shapes could be merged, despite the spectral variations in the segments.

Figure 3(b) shows kappa values for the classification with average color and shape weights of 0.5 (Step II), and 0.25 or 0.75 (Step III). Overall, the accuracies tended to decrease with increasing scale parameter, regardless of the color weight values.

When the color was weakly weighted and the shape was strongly weighted [C_1_S_9_(M_9_O_1_), C_1_S_9_(M_1_O_9_)], the accuracies tended to decrease dramatically as the scale parameter increased. The higher the weighting of the shape, the greater the decrease in the accuracies as the scale parameter increased. Segmentation with high weight for the shape parameter can cause the merging of segments of different tree species with different spectral values, leading to low segmentation accuracy by the kappa value.

**Figure 4.**
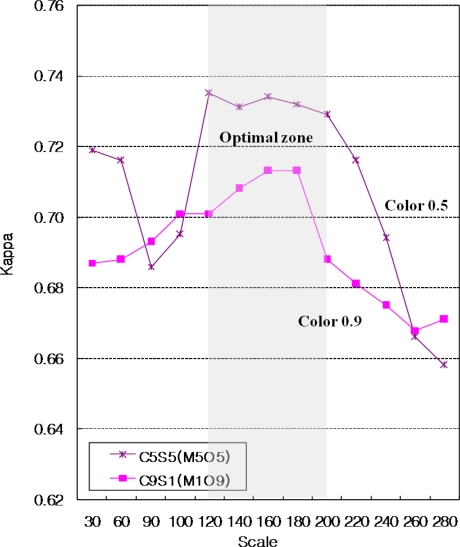
Optimal weights for segmentation.

Considering weight for color, the segmentation with strong weight for color was less sensitive to the scale parameter and had better accuracy. Our results demonstrated that segmentation with color parameters from 0.5 to 0.9 and scale parameter from 120 to 200 can lead to optimal forest cover segmentation and classification in central Korea with IKONOS satellite image (Figure 4).

### 3.3. Forest Cover Map

#### 3.3.1. PBC-Based Forest Cover Map

**Figure 5.**
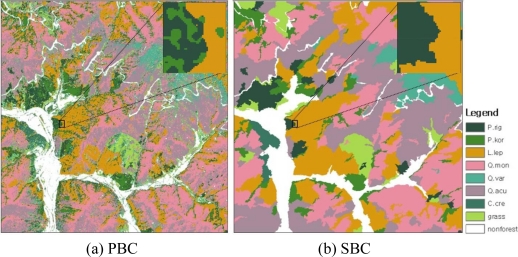
Forest cover map: **(a)** forest cover map of the pixel-based classification (PBC) using the maximum likelihood method, and **(b)** segment-based classification (SBC) with majority principle (color parameter of 0.5, scale parameter of 160).

Figure 5(a) shows the forest cover map produced by the PBC method. The high spatial resolution makes it possible to classify forest cover by tree species. However, the high spatial resolution revealed high variability of the pixel values within a particular class.

The conventional PBC with high spatial resolution image is inevitably associated with the “salt-and-pepper” effect, which results in small inclusion of other classes within a polygon [12]. A closer inspection of Figure 5 shows that the salt-and-pepper effect can be observed on the classification map. This salt-and-pepper effect makes it difficult to integrate the classification result into a polygon-based forest cover map. It has proven difficult to classify high-resolution images on a pixel-by-pixel basis due to the high level of information and increased intra-class complexity [31,34,39,40].

#### 3.3.2. SBC-Based Forest Cover Map

Several attempts to apply the object-based approach for classifying forest cover revealed the difficulty in formalizing the contextual, formal, spatial and structural relationships for the forest cover classification, as required in Definiens^®^ Professional 5.0.

In the SBC, a segmented image is classified by membership rule or maximum likelihood. Even though the SBC is an appropriate method for dividing the forest area, it results in misclassification due to the mean value of pixels in the segmented image. On the other hand, even though a precise classification with pixel units can be conducted by PBC, the gap or shadow in the forested area causes misclassification results. Therefore, in this study, when our method fuses the results of PBC and segment, it supplemented each weak point of PBC and SBC by applying the majority rule.

The SBC in this study produced a forest cover map without any salt-and-pepper effect (Figure 5(b)), as expected from the previous SBC [9,12,41,42]. Each error matrix for the SBC using a majority rule and PBC using maximum likelihood is shown in Tables 3 and 4. The use of the SBC avoided the salt-and-pepper effects of the PBC. In addition, the overall accuracy was improved from 64% in the PBC to 77% and the kappa value from 0.59 to 0.73. This detailed and reliable information about forest cover will be useful for several applications related to forestry planning and management, natural resources management, carbon cycle studies and for biogeochemistry, hydrology and climate monitoring [43].

#### 3.3.3. Comparison Analysis

For verifying the feasibleness of our methodology, the results were compared with previous methods that used the traditional majority rule-based filter with a 3 × 3 window and object-based approach. Objects were prepared using Definiens^®^ Professional 5 Program. The procedure of object-based classification (OBC) can achieve the same objective based on the segmentation of spectral bands of the image creating homogeneous polygons with regard to the single pixel value, shape, texture, and pixel spatial continuity [44].

**Figure 6.**
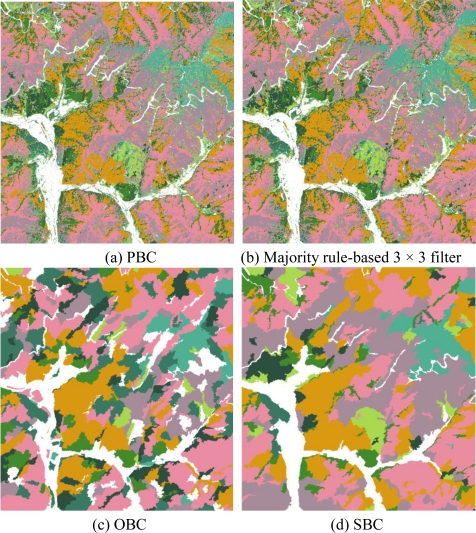
Forest cover maps generated by pixel-based (PBC), majority rule-based, object-based (OBC), with a color parameter of 0.5 and a scale parameter of 160 (C_5_S_5_(M_5_O_5_)), and segment-based (SBC) classification methods.

However, in this study, the accuracy of OBC was the lowest due to the over-segmentation when compared with other classification methods (Figure 6). This was attributed to the spectral similarity between the tree species by which the forest type might be misclassified, especially for *Pinus koraiensis* and *Pinus rigida*. Moreover, the sensitivity for stand shape and texture can lead to over-segmentation because of the various and irregular shapes of the forest stands (objects) in study area.

Majority rule-based 3 × 3 filter is one of the post-processing methods on PBC. Although these techniques can improve the classification accuracy considerably, their disadvantages are apparent when applied to high spatial resolution images (1 m to 10 m) [25]. This process suffers blur effects and cannot produce accurate results at the boundaries of different land-cover units. SBC reduces the local spectral variation caused by crown textures, gaps, and shadows. In addition, with spectrally homogeneous segments of images, both spectral values and spatial properties, such as size and shape, can be explicitly utilized as features for further classification. Therefore, the result of majority rule-based classification was analyzed to be lower than that of the segment-based method, despite being higher than other approaches.

## 4. Conclusions

This study investigated the suitability of high spatial resolution IKONOS images for preparing precise forest cover maps for central Korea. The experimental results demonstrated the potential capability of IKONOS images in discriminating forest cover types at the tree species level.

Segmentation weighted for shape was sensitive to the scale parameter, but tended to merge segments of different tree species with different spectral values. Based on a step-by-step analysis, this study suggested that segmentation, with color strongly weighted, was less sensitive to the scale parameter, leading to optimal forest cover segmentation and classification.

Pixel-based maximum likelihood classification produced an undesirable “salt-and-pepper” effect. SBC delivered higher accuracy and more homogeneous classification results that were free from the “salt-and-pepper” effect.

## Figures and Tables

**Table 1.** Classification schema for preparing forest cover map.

**Class description**	**Abbreviation**	**Common name**	**Large scale class**
*Pinus rigida*–stand	*P.rig*	Pitch pine	Forest area
*Pinus koraiensis*–stand	*P.kor*	Korean pine	
*Larix leptolepis*–stand	*L.lep*	Japanese larch	
*Quercus mongolica*–stand	*Q.mon*	Mongolian oak	
*Quercus variabilis*–stand	*Q.var*	Cork oak	
*Quercus acutissima*–stand	*Q.acu*	Sawtooth oak	
*Castanea crenata*–stand	*C.cre*	Chestnut	
Grass land	grass	-	

Agricultural area (dry, wet), Water, House, Road	Non-forest	-	Non-forest area

**Table 2.** Different weights for finding optimal segment size. The notation conveys information about the weights for color, shape, smoothness or compactness (C = color, S = shape, M = smoothness, O = compactness, subscript 1 = 0.1, 3 = 0.25, 5 = 0.5, 7 = 0.75 and 9 = 0.9).

**Step**	**Case**	**Color**	**Shape(smoothness/compactness)**
I	C_9_S_1_(M_9_O_1_)	0.9	0.1(0.9/0.1)
	C_9_S_1_(M_1_O_9_)	0.9	0.1(0.1/0.9)
	C_1_S_9_(M_9_O_1_)	0.1	0.9(0.9/0.1)
	C_1_S_9_(M_1_O_9_)	0.1	0.9(0.1/0.9)

II	C_5_S_5_(M_5_O_5_)	0.5	0.5(0.5/0.5)
	C_5_S_5_(M_9_O_1_)	0.5	0.5(0.9/0.1)
	C_5_S_5_(M_1_O_9_)	0.5	0.5(0.1/0.9)

III	C_7_S_3_(M_5_O_5_)	0.75	0.25(0.5/0.5)
	C_3_S_7_(M_5_O_5_)	0.25	0.75(0.5/0.5)

**Table 3.** Error matrix of the pixel-based classification (PBC) using a maximum likelihood method.

**Classes**		**Reference data**									**Sum**	**User’s Accuracy (%)**
		***P. rig***	***P. kor***	***L. lep***	***Q. mon***	***Q. var***	***Q. acu***	***C. cre***	**Grass**	**Non-forest**		
Classified data	*P. rig*	**13**	13	1	2	0	1	1	1	0	33	41.0
	*P. kor*	3	**25**	3	3	0	1	1	0	1	36	67.5
	*L. lep*	2	6	**40**	5	0	3	0	0	1	57	70.7
	*Q. mon*	0	1	3	**34**	1	10	0	0	1	49	67.9
	*Q. var*	0	1	1	2	**33**	5	1	4	5	53	63.0
	*Q. acu*	1	1	1	9	7	**27**	1	1	3	52	51.2
	*C. cre*	1	2	0	1	3	3	**14**	2	2	28	51.6
	Grass	1	1	2	1	4	4	2	**16**	3	35	47.2
	Non-forest	0	1	0	1	2	1	0	1	**59**	66	89.4

	Sum	22	50	53	57	51	56	20	26	74	**409**	
	
Producer’s Accuracy (%)		60.6	49.2	76.5	58.9	64.9	47.9	71.4	63.1	79.6	Overall accuracy: 63.9% Kappa value: 0.59	

**Table 4.** Error matrix of segment-based classification (SBC) using a majority rule, with a color parameter of 0.5 and a scale parameter of 160 (C_5_S_5_(M_5_O_5_)).

**Classes**		**Reference data**									**Sum**	**User’s Accuracy (%)**
		***P. rig***	***P. kor***	***L. lep***	***Q. mon***	***Q. var***	***Q. acu***	***C. cre***	**Grass**	**Non-forest**		
Classified data	*P. rig*	**19**	5	0	0	0	1	0	2	0	27	69.1
	*P. kor*	1	**28**	0	4	0	0	0	0	3	36	77.0
	*L. lep*	1	8	**44**	3	3	5	0	0	1	66	67.2
	*Q. mon*	0	4	7	**40**	0	4	1	0	0	56	71.9
	*Q. var*	0	1	0	1	**37**	1	0	0	0	40	92.0
	*Q. acu*	1	0	1	8	9	**44**	0	2	3	69	64.4
	*C. cre*	0	1	0	0	0	0	**18**	0	0	19	94.6
	Grass	0	2	0	0	2	1	0	**21**	4	31	69.0
	Non-forest	0	0	0	0	0	0	1	1	**63**	65	96.0

	Sum	22	50	53	57	51	56	20	26	74	**409**	
	
Producer's Accuracy (%)		84.8	55.5	84.5	70.8	72.0	78.9	88.5	81.4	84.7	Overall accuracy: 76.8%Kappa value: 0.73	

**Table 5.** Accuracy assessments by classification methods.

	**Overall accuracy (%)**	**Kappa value**
Pixel-based classification	63.9	0.59
Majority rule-based 3 × 3 filter	67.6	0.63
Object-based classification	53.1	0.42
Segment-based classification	76.8	0.73
